# The differential effects of fatty acids on enterocytic abundance of amyloid-beta

**DOI:** 10.1186/s12944-019-1162-9

**Published:** 2019-12-03

**Authors:** Susan Galloway, Ryusuke Takechi, Michael Nesbit, Menuka M. Pallebage-Gamarallage, Virginie Lam, John C. L. Mamo

**Affiliations:** 1 Curtin Health Innovation Research Institute, Curtin University, GPO Box U1987, Perth, WA 6845 Australia; 2 School of Public Health, Faculty of Health Sciences, Curtin University, GPO Box U1987, Perth, WA 6845 Australia

**Keywords:** Amyloid-β, Dietary intervention, Enterocytes, Saturated fatty acids, Small intestine, Unsaturated fatty acids

## Abstract

**Background:**

Consumption of a Western-styled diet enriched in saturated fatty acids (SFA) relative to polyunsaturated fatty acids is positively associated with risk for Alzheimer’s disease. Whilst potential causal mechanism are unclear, there is increasing evidence that chronic ingestion of SFA enriched diets promote increase the plasma levels of lipoprotein-associated amyloid-β (Aβ). However, the effects of dietary mono- and poly-unsaturated fats (MUFA/PUFA) on nascent lipoprotein Aβ abundance have not been previously reported.

**Methods:**

Wild-type C57BL/6 J mice were maintained on low-fat control chow (LF) or diets enriched in either SFA, MUFA, or PUFA for 9 months. Enterocytic abundance of Aβ was determined with quantitative immunofluorescent microscopy and plasma Aβ was measured by ELISA.

**Results:**

The chronic ingestion of SFA-enriched diet increased the enterocytic abundance and plasma concentration of Aβ compared to LF control mice. The mice maintained on MUFA or PUFA diet showed comparable enterocytic and plasma Aβ levels to the LF control mice.

**Conclusions:**

The data indicates that a diet enriched in SFA significantly increases the enterocytic Aβ production and secretion into the circulation, whilst MUFA and PUFA enriched diet do not exert such effects.

## Background

Alzheimer’s disease (AD) is the most prevalent form of dementia, accounting for more than 65% of all dementia cases. Epidemiological studies are consistent that consumption of dietary saturated fatty acids (SFA) is associated with an increased risk of AD. The Honolulu-Asia Aging study showed that Japanese men migrated to countries with greater dietary SFA intake showed a significantly higher risk of developing AD [1, 2]. A recent meta-analysis consistently also reported that a higher intake of dietary SFA increases the risk of AD by 39% [3]. On the other hand, mono- and poly-unsaturated fatty acids (MUFA/PUFA) are shown to have no significant association with AD risk and indeed, some studies suggest protective effects [3]. A number of epidemiological studies report that the frequent consumption of PUFA containing fish reduces the risk of AD [4]. Studies from France, Italy and Australia indicated that the intake of MUFA prevents cognitive decline and by extension AD [5–7]. However, the exact mechanisms how dietary fatty acids differentially modulate the AD risk are presently unclear.

The hallmark pathophysiological feature of AD is amyloid plaques, formed as a result of insoluble aggregation of amyloid-β (Aβ) peptides. Whilst the origin of Aβ that is deposited in the AD brain parenchyme has not been made clear to date, substantial amount of Aβ is found in the peripheral body [8]. An emerging line of evidence is consistent that the peripherally derived Aβ makes significant contribution to the cerebral amyloidosis in addition to the Aβ of CNS origin [9–11]. Furthermore, a recent study demonstrated that the plasma concentration of soluble Aβ correlates with the cerebral Aβ burden in both healthy and Alzheimer’s patients with approximately 90% accuracy [12].

Matsubara et al. reported that in the peripheral circulation, more than 97% of soluble Aβ is bound to lipoprotein particles [13]. Our previous study revealed that of those lipoprotein-bound Aβ in plasma, approximately 60% is associated with triglyceride rich lipoproteins (TRLs), that are of intestinal and hepatic origin [14]. We also reported that such lipogenic organs can synthesize and secrete Aβ complexed to lipoproteins into the circulation [15–18]. Moreover, the synthesis of Aβ within the small intestinal epithelial cells was regulated by the ingestion of dietary SFA [17]. In wild-type C57BL/6 mice, the chronic feeding of SFA significantly increased the enterocytic production of Aβ compared to the mice that were maintained on low-fat standard chow, whilst the fasting of the mice for 12 h completely abolished the enterocytic Aβ [16]. These data consistently suggest that different types of fatty acids may differentially influence the postprandial production and secretion of TRL-associated Aβ, and by extension modulate plasma Aβ homeostasis. However, to date, the effects of unsaturated fatty acids on enterocytic Aβ abundance and secretion into the circulation have not been reported. Therefore, in this study, we have tested the effects of chronic MUFA and PUFA feeding in comparison to SFA on the abundance of enterocytic and plasma Aβ.

## Methods

### Animals and dietary intervention

Six week-old female wild-type C57BL/6 J mice were obtained from the Animal Resources Centre (WA, Australia). Animals were randomly divided into groups (*n* = 12 per group): LF (low fat), SFA (saturated fatty acid), MUFA (monounsaturated fatty acid) or PUFA (polyunsaturated fatty acid) group. Experimental diets were manufactured by Specialty Feeds (WA, Australia). LF diet was AIN93M (15.1 MJ/Kg, 4% fat (w/w) see Table 1 for detail). High fat diets (18.8 MJ/Kg) were made by addition fats from different sources to AIN93M. The SFA chow containing 23% (w/w) fat from cocoa butter (SF07–050) predominantly contained 5.16% palmitic acid (16:0) and 7.31% stearic acid (18:0) and also 6.62% monounsaturated fat as oleic acid. The MUFA chow containing 23% fat from Sunola oil (SF07–051) contained 15.7% oleic acid, 2.4% linoleic acid (18:2 n6) and trace amounts of other fats. The PUFA chow containing 23% fat from NUMEGA fish oil (SF07–049) contained 8.2% docosahexaenoic acid (DHA 22:6 n3), 2.0% eicosapentaemoic acid (EPA 20:5 n3) with a high n-3/n-6 ratio of 13.4. The PUFA diet also contained 3.26% palmitic acid and 2.25% oleic acid. All fatty acids enriched diets contained reduced amount of wheat starch in order to reduce the overall energy of the diet per weight. For more details, see Table 1. Animals were contained in an environment which was controlled for temperature, air pressure and lighting (12:12 h light/dark cycles). Mice had access to food and water ad-libitum and remained on diets for periods of 9 months. Procedures relating to mice handling and sacrifice were performed in accordance with the Animal Ethics Committee Guidelines (Curtin University ethics approval no. R 02–07).

**Table 1:** Details of diets

Ingredients (g/kg)	LF	SFA	MUFA	PUFA
Casein	140	140	140	140
DL Methionine	1.8	1.8	1.8	1.8
Sucrose	100	100	100	100
Wheat Starch	472	308	308	308
Dextrinised starch	155	155	155	155
cellulose	50	50	50	50
Calcium carbonate	13.1	13.1	13.1	13.1
Sodium chloride	2.6	2.6	2.6	2.6
Potassium citrate	1	1	1	1
Potassium dihydrogen phosphate	8.8	8.8	8.8	8.8
Potassium sulphate	1.6	1.6	1.6	1.6
AIN93G trace minerals	1.4	1.4	1.4	1.4
Choline chloride (65%)	2.5	2.5	2.5	2.5
AIN93G vitamins	10	10	10	10
Canola oil	40	0	0	0
Cocoa butter	0	204	0	0
Sunol oil	0	0	204	0
NUMEGA fish oil	0	0	0	204
Lipid content (%)	LF	SFA	MUFA	PUFA
Myristic acid (14:0)	n/d	0.05	0.02	0.54
Pentadecanoic acid (15:0)	n/d	0.01	n/d	0.16
Palmitic acid (16:0)	0.2	5.16	0.85	3.26
Magaric acid (17:0)	n/d	0.05	n/d	0.18
Stearic acid (18:0)	0.1	7.31	0.87	0.92
Arachidic acid (20:0)	n/d	0.24	n/d	0.06
Behenic acid (22:0)	n/d	0.04	n/d	n/d
Tetracosanoic acid (24:0)	n/d	0.03	n/d	n/d
Palmitoleic acid (16:1)	trace	0.05	0.02	0.66
Heptadecenoic acid (17:1)	n/d	0.01	n/d	0.1
Oleic acid (18:1 n-9)	2.4	6.62	15.7	2.25
Gadoleic acid (20:1)	trace	0.01	0.07	0.18
Linoleic acid (18:2 n-6)	0.8	0.67	2.42	0.23
alpha Linolenic acid (18:3 n-3)	0.4	0.05	0.13	0.09
gamma Linolenic acid (18:3 n-6)	n/d	n/d	n/d	0.08
Stearidonicacid (18:4 n-3)	n/d	n/d	0.08	n/d
Arachadonic acid (20:4 n-6)	trace	n/d	0.2	0.46
EPA (20:5 n-3)	trace	n/d	n/d	2
DPA (22:5 n-3)	n/d	n/d	n/d	0.3
DHA (22:6 n-3)	trace	n/d	n/d	8.22

*LF* Low-fat control; *SFA* saturated fatty acids; *MUFA* monounsaturated fatty acids; *PUFA* polyunsaturated fatty acids

### Sample collection

Mice from each dietary intervention group were sacrificed at 9 months. Mice were anaesthetized with an intraperitoneal injection of Phenobarbital (45 mg/kg). Blood was collected by cardiac puncture into ethylene-diamine-tetracetic acid (EDTA)-tubes. For intestinal Aβ immunofluorescence, the digestive tract was removed and small intestinal the length of the small intestines were flushed with chilled phosphate buffered saline (PBS, pH = 7.4) and 2 cm was cut and removed for fixation in 4% paraformaldehyde. Tissues were fixed for 24 h and processed and longitudinal sections were embedded into paraffin wax blocks. Sections were trimmed to where all villi were exposed. Five-micron serial sections were cut and mounted on silanised-coated slides for immunostaining and histology.

### Immunofluorescent detection of enterocytic Aβ

Analysis of intestinal Aβ using immunofluorescent microscopy was done as previously described [17, 19]. In brief, 5-μm thick sections were deparaffinised and rehydrated and then placed in boiling deionised water for 15 mins to retrieve antigens and for a further 10 mins in PBS with Tween-20 to permeabilise tissues before blocking in 20% goat serum. Rabbit anti-human Aβ (Chemicon Temecula, CA) was added to slides and allowed to incubate overnight at 4 °C. Immunofluorescence was visualised with addition of anti-rabbit IgG with Alexa488 (1:100). Cell nuclei were labelled with DAPI (1:1000) (Invitrogen, USA).

3-D confocal immunofluorescent digital images were captured with UltraVIEW Vox microscopy (PerkinElmer, UK) with 40x objective lens. For sufficient statistical power, approximately 25 images were taken randomly from each intestinal section per mouse by a blinded investigator. The voxel intensity of Aβ staining within the small intestinal enterocytes was analysed by using Zen Intellesis and Measurement image analysis software modules (Zeiss, Germany).

### Plasma Aβ analysis

Plasma Aβ was measured using a commercially available ELISA kits which detects mouse Aβ40 and Aβ42 (Invitrogen). ELISA immunodetection method was performed as per the instructions of the manufacturer. The plasma concentrations of Aβ40 and Aβ42 were determined against the standards of Aβ40 ranging 0–500 pg/ml and Aβ42 ranging 0–200 pg/ml. The data is shown as combined Aβ40 and Aβ42.

### Plasma apolipoprotein B analysis

An exclusive, surrogate marker of TRLs, apolipoprotein (apo) B was determined in mouse plasma by Western blots as previously described [15]. Briefly, apoB100 and apoB48 in the plasma samples were separated on SDS-PAGE using 3–8% tris-acetate gel (Invitrogen) and electrotransferred onto PVDF membrane. Following the blocking with 5% skim milk, the membrane was incubated with rabbit anti-mouse apoB (Abcam, UK), and subsequently with anti-rabbit IgG conjugated with horseradish peroxidase. The protein bands for apoB was visualized with enhanced chemiluminescence. The apoB100 and B48 bands were quantified against a standard apoB peptides by using ImageJ. Sum of apoB100 and B48 is presented.

### Plasma lipid analysis

Plasma concentrations of cholesterol and triglycerides were measured with commercial colorimetric kits (Randox Laboratories, UK) according to the manufacturer’s instruction manual. Briefly, 2 μl standards or plasma samples were mixed with 200 μl reaction solution on 96-well microplates, and incubated at 37 °C for 5 min. Subsequently, the absorbance was read at 550 nm and the lipids concentrations were extrapolated against the standard curve.

### Statistical analysis

All data are normally distributed and are expressed as mean ± SEM. The data was statistically analysed with one-way ANOVA followed by Fisher’s LSD post-hoc test and *p* < 0.05 was considered as statistically significant (GraphPad Prism).

## Results

**Fig. 1:**
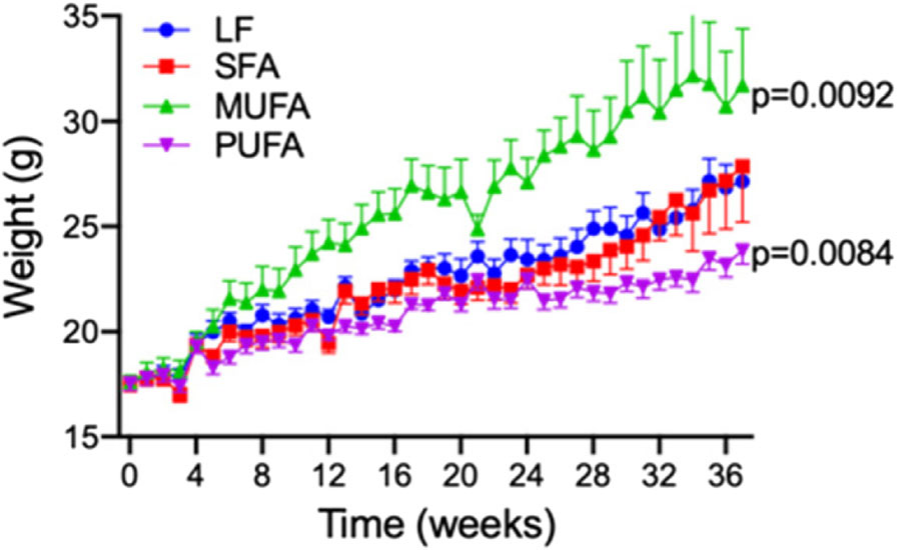
Animal weights. Weekly weight changes of C57BL6J mice that were maintained on either low-fat control chow (LF), diet enriched in saturated fatty acids (SFA), monounsaturated fatty acids (MUFA) or polyunsaturated fatty acids (PUFA). Statistical significance was determined with one-way ANOVA with Fisher’s LSD post-hoc test (*n* = 12, p values indicated only for significance)

The diets were well tolerated and all mice gained weight consistently each week throughout the course of experiment. At the end of the experiment (9 months), the MUFA group gained significantly greater weight than other treatment groups. Conversely, the PUFA treated mice gained significantly less weight than other fat supplemented treatment groups at the end of the dietary regimen (Fig. 1). Average chow consumption was not different between all fatty acids groups (LF: 3.41 g SFA: 3.12 g, MUFA: 3.38 g, PUFA: 3.32 g).

**Fig. 2:**
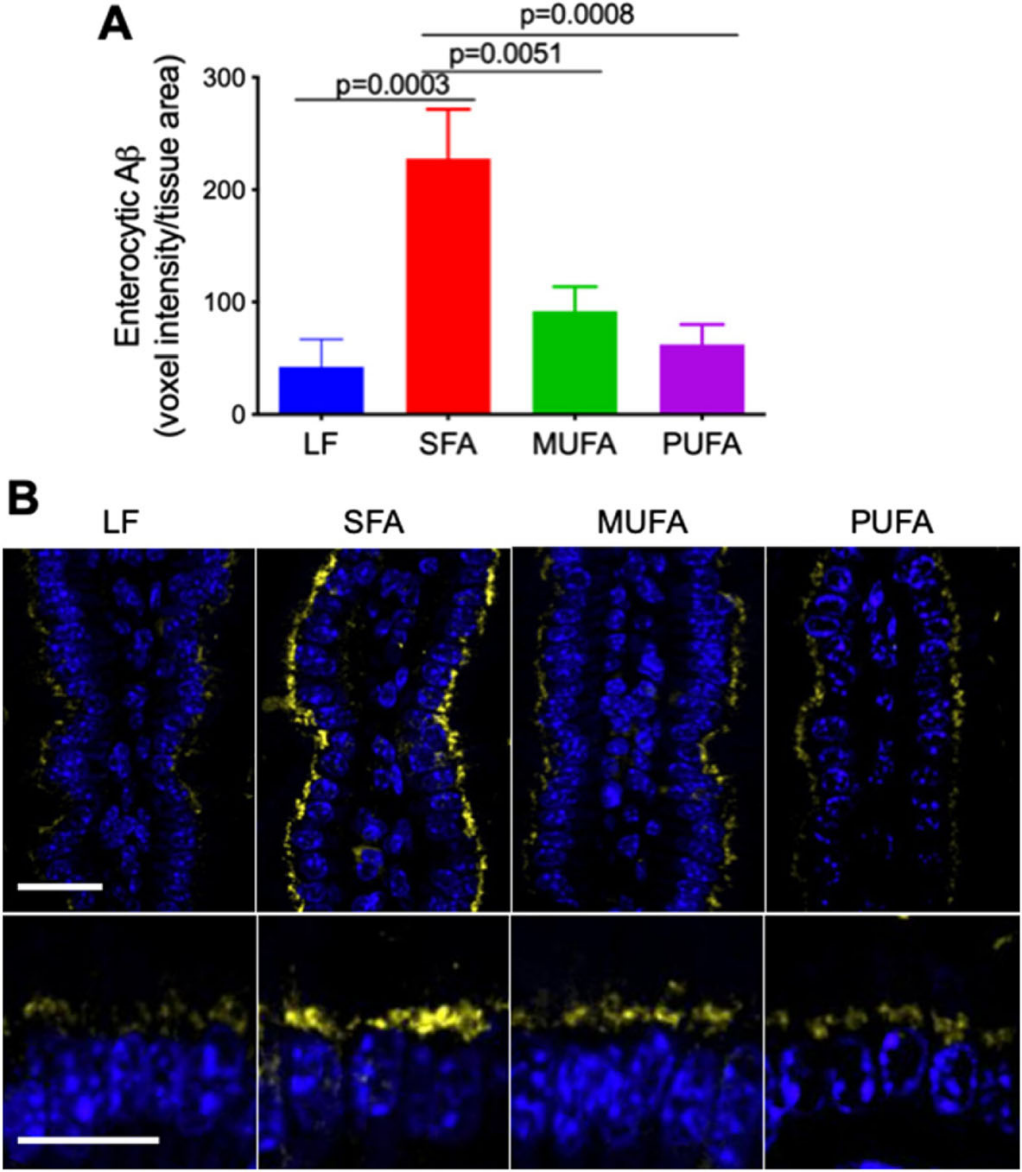
Intestinal abundance of Aβ. The abundance of Aβ in the mice maintained on low-fat control chow (LF), diet enriched in saturated fatty acids (SFA), monounsaturated fatty acids (MUFA) or polyunsaturated fatty acids (PUFA) was semi-quantitatively determined with immunofluorescent microscopy. **a** Semi-quantitative abundance of Aβ is expressed as voxel intensity of immunoreactivity per tissue area. Statistical significance was estimated with one-way ANOVA with Fisher’s LSD post-hoc test (*n* = 12, *p* values indicated only for significance). **b** Representative microscopy images are shown at low magnification (top row; scale bar = 5 μm) and high magnification (bottom row; scale bar = 5 μm). Aβ immunoreactivity is shown in yellow, and the nuclei is in blue

Following 9 months of dietary intervention with the SFA enriched diet, the enterocytic abundance of Aβ was significantly elevated compared to control mice maintained on a LF chow (Fig. 2). The mice fed with MUFA diet for 9 months showed no significant changes in enterocytic Aβ compared to LF control mice, but rather was substantially lower than the SFA-fed mice. Similarly, the enterocytic Aβ in mice maintained on PUFA diet for 9 months was comparable to control and MUFA mice.

**Fig. 3:**
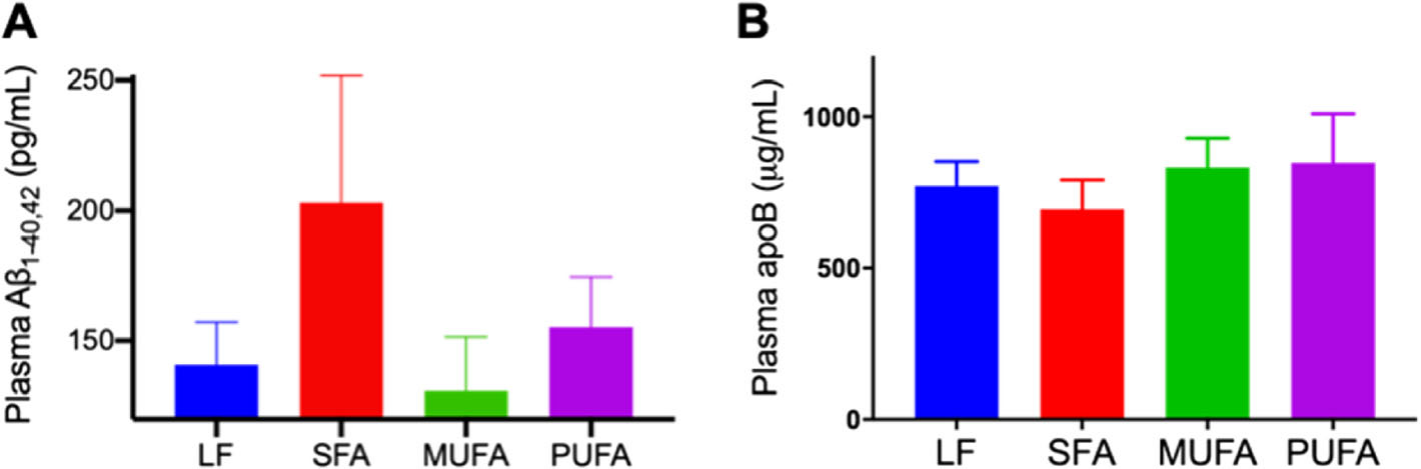
Plasma concentrations of Aβ and apolipoprotein B. **a** The concentrations of Aβ_1–40,42_ in plasma of the mice fed with either low-fat control chow (LF), diet enriched in saturated fatty acids (SFA), monounsaturated fatty acids (MUFA) or polyunsaturated fatty acids (PUFA) were measured with commercially available ELISA kits. **b** The plasma levels of apolipoprotein B (apoB) were determined with gel electrophoresis and Western blot. Statistical significance was assessed with one-way ANOVA with Fisher’s LSD post-hoc test (*n* = 12, no significance detected at *p* < 0.05)

The plasma abundance of Aβ in mice was determined by ELISA and is indicated in Fig. 3a. The net abundance of Aβ_1–40,42_ was more than 40% greater in SFA mice compared to LF, MUFA or PUFA treatment groups, although it did not reach statistical significance.

The analysis of plasma apoB, an exclusive marker of intestinal and hepatically derived TRLs with Western blot showed no significant changes in total apoB levels between the mice that were maintained on LF control and SFA diets for 9 months (Fig. 3b). Comparable levels of plasma apoB was also observed in the mice maintained on MUFA and PUFA diets for 9 months compared to LF control or SFA mice. Similarly, the plasma levels of cholesterol and triglycerides were not changed in the mice fed with SFA diet, compared to the LF control mice (Table 2). The plasma lipid levels were also comparable in MUFA and PUFA mice compared to LF or SFA mice.

**Table 2:** Plasma Lipids

	Cholesterol (mmol/L)	Triglyceride (mmol/L)
LF	1.84 ± 0.075	0.64 ± 0.056
SFA	2.23 ± 0.274	0.72 ± 0.125
MUFA	2.30 ± 0.312	0.67 ± 0.095
PUFA	2.57 ± 0.164	0.74 ± 0.105

*LF* Low-fat control; *SFA* saturated fatty acids; *MUFA* monounsaturated fatty acids; *PUFA* polyunsaturated fatty acids

## Discussion

This study investigated the effects of diets enriched in different types of fatty acids on the enterocytic Aβ and plasma Aβ.

Consistent with the previous observations [16], wild-type C57BL/6 mice maintained on a control LF chow for 9 months showed positive immunoreactivity of Aβ within the perinuclear region of small intestinal enterocytes. After a chronic ingestion of a diet that is enriched in SFA for 9 months, the enterocytic abundance of Aβ was significantly increased compared to the LF control mice, indicating the increased synthesis of Aβ by the small intestine in response to exogenous dietary SFA ingestion. The finding is consistent with our previous studies showing significant elevation of enterocytic Aβ following chronic ingestion of SFA-enriched diets [16, 20, 21].

The plasma concentration of Aβ in SFA-fed mice suggested an exaggerated post-prandial response in mice maintained on the SFA diet, compared to mice provided a diet enriched in LF chow. Dietary SFA-induced effects on plasma postprandial lipoprotein Aβ homeostasis was not realised as an accumulation of apoB lipoproteins per se and indeed SFA treated mice were normolipidemic. Rather, the findings suggest that dietary SFA result in significant enrichment of nascent lipoproteins with Aβ. Consistent with the functional role of apoproteins [22], it is a reasonable proposition that the latter could alter catabolism and/or cellular function.

In the present study, we also report for the first time, the effects of MUFA and PUFA enriched diets on small intestinal abundance and plasma concentrations of Aβ. After 9 months feeding of a diet enriched in MUFA, the mice showed no significant increase of Aβ in the small intestinal enterocytes compared to the mice maintained on LF control chow for 9 months. Consistent with this observation, plasma concentrations of Aβ remained unchanged in the MUFA-fed mice compared to the LF mice. Furthermore, no changes to the plasma apoB levels were observed in the mice maintained on MUFA diet, indicating that the chronic ingestion of MUFA-enriched diet did not significantly affect the enterocytic production of TRL-associated Aβ, or its circulating levels in wild-type mice. Similarly, the mice that were fed a diet enriched in PUFA for 9 months showed comparable enterocytic Aβ abundance to the control mice fed LF diet. In addition, the plasma concentrations of Aβ and apoB remained unchanged, indicating that PUFA-enriched diet also did not significantly impact on enterocytic Aβ production or circulating Aβ. These findings suggest that in contrast to SFA, the chronic ingestion of equicaloric unsaturated fats does not result in the increase of enterocytic Aβ production and thereafter the secretion of TRL-Aβ complex into the circulation. This aligns with the previously reported beneficial effects of MUFA and PUFA on AD risks. Indeed, Gu et al. found that in a cross-sectional study of 1219 cognitively healthy elderly individuals, higher intake of PUFA relative to SFA was strongly associated with lower plasma Aβ levels [23]. The findings of the present study may partially explain the underlying mechanisms by which PUFA protects against AD and lowers plasma Aβ.

The limitation of the study was that it does not examine the effects of specific fatty acid types on the peripheral Aβ, but rather considers the ‘SFA-enriched dietary behaviour’ and ‘low-fat healthy lifestyle’. The future studies may be necessary to investigate specifically which components of the SFA, MUFA or PUFA diet have effects on the intestinal Aβ.

## Conclusions

The present study for the first time reports that the chronic ingestion of MUFA or PUFA enriched diet does not influence the intestinal abundance Aβ and plasma concentrations of TRL-Aβ. Consistent with previous observations, SFA-enriched diet significantly increased the enterocytic synthesis and secretion of Aβ as lipoprotein complex. The findings may provide important mechanistic insights for the differential effects of dietary fatty acids intake on AD risks.

## Data Availability

All data generated or analysed during this study are included in this published article.

## Abbreviations

AD: Alzheimer’s disease
Aβ: Amyloid-β
LF: Low fat
MUFA: Monounsaturated fatty acids
PUFA: Polyunsaturated fatty acids
SFA: Saturated fatty acids
TRL: Triglyceride rich lipoprotein

## References

[1] Shadlen MF, Larson EB, Yukawa M (2000). The epidemiology of Alzheimer's disease and vascular dementia in Japanese and African-American populations: the search for etiological clues. Neurobiol Aging.

[2] Havlik RJ, Izmirlian G, Petrovitch H, Ross GW, Masaki K, Curb JD (2000). APOE-epsilon4 predicts incident AD in Japanese-American men: the Honolulu-asia aging study. Neurology..

[3] Ruan Y, Tang J, Guo X, Li K, Li D (2018). Dietary fat intake and risk of Alzheimer's disease and dementia: a meta-analysis of cohort studies. Curr Alzheimer Res.

[4] van Gelder BM, Tijhuis M, Kalmijn S, Kromhout D (2007). Fish consumption, n-3 fatty acids, and subsequent 5-y cognitive decline in elderly men: the Zutphen elderly study. Am J Clin Nutr.

[5] Tangney CC, Kwasny MJ, Li H, Wilson RS, Evans DA, Morris MC (2011). Adherence to a Mediterranean-type dietary pattern and cognitive decline in a community population. Am J Clin Nutr.

[6] Panza F, Frisardi V, Seripa D, Imbimbo BP, Pilotto A, Solfrizzi V (2010). Dietary unsaturated fatty acids and risk of mild cognitive impairment. J Alzheimers Dis.

[7] Gardener S, Gu Y, Rainey-Smith SR, Keogh JB, Clifton PM, Mathieson SL (2012). Adherence to a Mediterranean diet and Alzheimer's disease risk in an Australian population. Transl Psychiatry.

[8] Piccarducci R, Pietrobono D, Pellegrini C, Daniele S, Fornai M, Antonioli L (2019). High levels of beta-amyloid, tau, and Phospho-tau in red blood cells as biomarkers of neuropathology in senescence-accelerated mouse. Oxidative Med Cell Longev.

[9] Takechi R, Galloway S, Pallebage-Gamarallage MM, Lam V, Mamo JC (2010). Dietary fats, cerebrovasculature integrity and Alzheimer's disease risk. Prog Lipid Res.

[10] Takechi R, Galloway S, Pallebage-Gamarallage MM, Wellington CL, Johnsen RD, Dhaliwal SS (2010). Differential effects of dietary fatty acids on the cerebral distribution of plasma-derived apo B lipoproteins with amyloid-beta. Br J Nutr.

[11] Takechi R, Galloway S, Pallebage-Gamarallage M, Wellington C, Johnsen R, Mamo JC (2009). Three-dimensional colocalization analysis of plasma-derived apolipoprotein B with amyloid plaques in APP/PS1 transgenic mice. Histochem Cell Biol.

[12] Nakamura A, Kaneko N, Villemagne VL, Kato T, Doecke J, Dore V (2018). High performance plasma amyloid-beta biomarkers for Alzheimer's disease. Nature..

[13] Matsubara E, Sekijima Y, Tokuda T, Urakami K, Amari M, Shizuka-Ikeda M (2004). Soluble Abeta homeostasis in AD and DS: impairment of anti-amyloidogenic protection by lipoproteins. Neurobiol Aging.

[14] Mamo JC, Jian L, James AP, Flicker L, Esselmann H, Wiltfang J (2008). Plasma lipoprotein beta-amyloid in subjects with Alzheimer's disease or mild cognitive impairment. Ann Clin Biochem.

[15] Galloway S, Takechi R, Pallebage-Gamarallage MM, Dhaliwal SS, Mamo JC (2009). Amyloid-beta colocalizes with apolipoprotein B in absorptive cells of the small intestine. Lipids Health Dis.

[16] Galloway S, Jian L, Johnsen R, Chew S, Mamo JC (2007). Beta-amyloid or its precursor protein is found in epithelial cells of the small intestine and is stimulated by high-fat feeding. J Nutr Biochem.

[17] Pallebage-Gamarallage MM, Galloway S, Takechi R, Dhaliwal S, Mamo JC (2012). Probucol suppresses enterocytic accumulation of amyloid-beta induced by saturated fat and cholesterol feeding. Lipids.

[18] Koudinov AR, Koudinova NV (1997). Alzheimer's soluble amyloid beta protein is secreted by HepG2 cells as an apolipoprotein. Cell Biol Int.

[19] Takechi R, Galloway S, Pallebage-Gamarallage MM, Johnsen RD, Mamo JC (2008). Three-dimensional immunofluorescent double labelling using polyclonal antibodies derived from the same species: enterocytic colocalization of chylomicrons with Golgi apparatus. Histochem Cell Biol.

[20] Takechi R, Pallebage-Gamarallage MM, Lam V, Giles C, Mamo JC (2014). Long-term probucol therapy continues to suppress markers of neurovascular inflammation in a dietary induced model of cerebral capillary dysfunction. Lipids Health Dis.

[21] Takechi R, Galloway S, Pallebage-Gamarallage MM, Mamo JC (2008). Chylomicron amyloid-beta in the aetiology of Alzheimer's disease. Atheroscler Suppl.

[22] James AP, Pal S, Gennat HC, Vine DF, Mamo JC (2003). The incorporation and metabolism of amyloid-beta into chylomicron-like lipid emulsions. J Alzheimers Dis.

[23] Gu Y, Schupf N, Cosentino SA, Luchsinger JA, Scarmeas N (2012). Nutrient intake and plasma beta-amyloid. Neurology.

